# Feasibility and preliminary efficacy of an online mindful walking intervention among COVID-19 long haulers: A mixed methods study including daily diary surveys

**DOI:** 10.1371/journal.pdig.0000794

**Published:** 2025-04-08

**Authors:** Abhishek Aggarwal, Shan Qiao, Chih-Hsiang Yang, Slone Taylor, Cheuk Chi Tam, Xiaoming Li

**Affiliations:** 1 Department of Health Promotion Education and Behavior, Arnold School of Public Health, University of South Carolina, Columbia, United States of America; 2 South Carolina SmartState Center for Healthcare Quality, Arnold School of Public Health, University of South Carolina, Columbia, United States of America; 3 Exercise Science Department, Arnold School of Public Health, University of South Carolina, Columbia, United States of America; 4 Department of Epidemiology and Biostatistics, Arnold School of Public Health, University of South Carolina, Columbia, United States of America; Iran University of Medical Sciences, ISLAMIC REPUBLIC OF IRAN

## Abstract

COVID-19 long haulers face profound psychosocial stressors (e.g., depression, anxiety, PTSD) and physical health challenges (e.g., brain fog, fatigue). This study tests the feasibility and initial impact of a digitally delivered mindful-walking (MW) intervention for improving the physical and psychosocial wellbeing of COVID-19 long haulers. We recruited 23 participants via Facebook groups, between March and November 2021, for a 4-week online MW intervention (i.e., 2 mindfulness practice sessions per week), that was delivered entirely through the study Facebook group. The intervention was assessed using mixed methods. Quantitative data were collected through brief daily evening surveys (i.e., 28 days) over the 4-week intervention period, and measured affect, cognition, mindfulness, physical activity, and MW engagement. Qualitative data were extracted from the Facebook group’s Paradata (i.e., participant feedback, engagement metrics, and all social media interactions). Multilevel modeling was employed for the statistical analysis and a pragmatic approach was used for the qualitative analysis. The participants reported a high feasibility score (mean=4.93/7, SD=1.88), which was comprised of perceived usefulness, satisfaction, and ease of use. Those who engaged in MW, on any given day, frequently reported better psychosocial moods with more positive affect (β=0.89, p<0.01), less negative affect (β=−0.83, p<0.01), higher perceived cognitive ability (β=0.52, p<0.05), and more physical activity (β=0.41, p<0.05). Additionally, participants who practiced MW more consistently during the study reported higher levels of momentary mindfulness (β=0.3 p<0.01). Participants expressed satisfaction with the intervention, reporting benefits such as better symptom management and an overall improvement in wellbeing. Despite the small sample size, the digital delivery of our MW intervention via Facebook showed high acceptability. Preliminary efficacy findings indicate improved mental wellbeing and physical activity among long haulers. Larger-scale RCTs are needed in the future to improve the robustness and applicability of findings.

## Author summary

In this study, we investigated the feasibility of delivering a mindful-walking (MW) intervention to COVID-19 long haulers, via social media, and assessed its preliminary efficacy on enhancing their physical and psychosocial wellbeing. COVID-19 long haulers often experience lasting physical and emotional challenges, and MW may be an effective strategy to address these issues. We recruited participants from Facebook groups and conducted a 4-week MW program, where mindfulness sessions were administered twice a week. Participants completed daily surveys on their affect, cognition, mindfulness, physical activity, and MW engagement. Our findings showed that those who engaged in MW, on any given day, frequently reported better affect, perceived cognition, and physical activity levels. Additionally, participants who practiced MW more consistently during the study reported higher levels of momentary mindfulness. Participants expressed satisfaction with the intervention, noting benefits such as better symptom management and an overall improvement in wellbeing. These results suggest that a digital MW program can be a valuable tool for improving the mental and physical health of COVID-19 long haulers.

## Introduction

COVID-19 has left a significant proportion of patients, termed “long haulers,” grappling with persistent symptoms such as chronic fatigue, cognitive issues, and loss of taste and smell that hinder their physical and psychosocial wellbeing [1–3]. Long haulers have been found to experience ongoing cognitive and psychiatric challenges, including PTSD and depression [4,5], alongside physical symptoms such as fatigue and respiratory difficulties [6]. These issues often lead to socio-economic impacts, including social isolation and financial instability [7–9]. Notably, existing data have shown that long haulers may exhibit behaviors of substance misuse to manage their cognitive and psychiatric challenges [10], which increases the risk for additional health issues, such as morbidity and mortality associated with substance use. Given these multifaceted challenges, a psycho-behavioral health promotion intervention addressing the quality of life among long haulers is warranted.

Outdoor physical activity holds strong potential as an effective coping and preventive strategy, for long COVID (i.e., sustained COVID-19 symptoms experienced by long haulers), given its well-documented physical, social, and mental health benefits for people of all ages, especially those with or at risk of developing chronic diseases [11]. A recent cross-sectional study reported that physical activity during the pandemic was associated with a reduced likelihood of long COVID and a reduced duration of long COVID symptoms, including fatigue, neurological complications, cough, and loss of sense of smell or taste [12]. Therefore, physical activity can play a crucial role in the recovery of long haulers, including regaining their physical stamina post-illness [13].

A mindfulness practice may be a promising component of health interventions specifically targeting long haulers to address physical and psychosocial wellbeing challenges as well as to reduce distress associated with various stressors. Mindfulness highlights the connection between physical and sensational cues for mental health promotion. As highlighted in several systematic reviews, mindfulness can effectively reduce anxiety, depression, and distress in patients with chronic diseases, including cancer and diabetes [14–16]. Combining mindfulness and physical activity has the potential to enhance physical activity levels by fostering greater awareness and attention to present experiences, thereby promoting a more active lifestyle [17–18]. Mindful walking (MW) is a mindfulness practice that is preventive, self-initiated, easily integrated into daily routines, and less resource-intensive while offering comparable benefits to other forms of physical activity [19].

Integrating mindfulness techniques with routine physical activities, such as walking, can enhance state mindfulness while promoting physical activity [20]. MW is an accessible and low-intensity exercise that could gently rebuild the physical strength and endurance of long haulers, without over exertion, and could assist in managing the stress and anxiety associated with the recovery process [21–23]. MW combines low-intensity physical activity with mindfulness, fostering both physical recovery and mental wellbeing through heightened awareness of bodily sensations and mental states. This dual focus makes it a promising intervention for long haulers seeking gradual physical reconditioning and mental stability. Hence, MW may foster both physical and mental rehabilitation for long haulers, in alignment with their health goals, by easily fitting into daily life routines for sustained practice.

A growing number of interventions have been developed to address the wellbeing of long haulers. A comprehensive scoping review highlighted a range of interventions from singular approaches like pharmacological treatments, electromagnetic field sessions, and dietary modifications, to holistic programs encompassing multiple health components [24]. These integrated programs touched upon aspects such as rehabilitation, lifestyle changes, stress management, sleep hygiene, breathing techniques, dietary optimization, energy conservation, psychoeducation, maintaining social interactions, and cognitive behavioral therapy (CBT). While some interventions emphasized either mindfulness practice or physical activity, few combined both [25]. No MW interventions targeted long haulers [25]. This observation underscores a clear gap in the literature and demonstrates the importance of evaluating the feasibility and preliminary efficacy of a MW program among long haulers.

In the context of the COVID-19 pandemic, the adoption of online platforms for healthcare interventions has become indispensable. Social media platforms enable accessible, flexible, and socially supportive environments for behavioral interventions, particularly for individuals facing mobility constraints due to health conditions. Social media, or online social networking sites (e.g., Facebook, Twitter, Reddit, Pinterest, Instagram), provide a means for users to engage with each other and share content, presenting an opportunity to use this modality for behavior change interventions. Social media usage has been stable over the past 5 years, with 7 in 10 US adults reporting using any kind of social media platform [26]. Particularly, Facebook remains a dominate platform with 69% of US adults reporting its use [26]. The use of social media has evolved, with more users now seeking and exchanging health-related information across platforms [27]. The pandemic’s physical distancing mandates, transportation barriers, and inaccessibility to traditional healthcare facilities [28] have underscored the importance of such online platforms for interventions. Delivering behavioral interventions via a social media platform eliminates the need for physical visits, offers flexibility in participation timing, integrates interventions seamlessly into daily routines, and readily offers access to social resources [29].

Online mindfulness interventions have been effective in decreasing depression, anxiety, stress, and improving mindfulness among patients with physical health conditions (e.g., cancer, chronic pain, heart disease) [30–32]. A prior pilot study with college students in China demonstrated the potential of a MW intervention, delivered via a smartphone app, showing initial effectiveness in improving mental health [22]. However, to date, no studies have explored the feasibility and preliminary efficacy of delivering a social media-based MW intervention for long haulers [33].

### The present study

Despite the promising findings of the individual benefits of mindfulness and physical activity, no study has yet explored the feasibility and preliminary efficacy of a MW intervention tailored for long haulers, particularly through a digital medium such as social media. In our study, we aim to pilot-test a MW program, delivered through a social media platform, for long haulers. Our approach includes administering daily diary surveys (i.e., to measure participants’ day-level psychological and physical activity outcomes), a one-month follow-up survey post-intervention (i.e., to assess the feasibility of digitally delivering the MW intervention), and qualitative data extracted from the study’s social media page (i.e., for a richer understanding of the participants’ personal experiences and feedback). This mixed-methods approach is designed not only to evaluate the program’s immediate effects but also to inform refinements for future iterations, ensuring sustained engagement and maximal benefits for long haulers.

## Materials and methods

### Recruitment

Participant recruitment was conducted over 7 months, from the end of March 2021 to the end of October 2021. Purposive sampling was employed to recruit participants primarily through the social media platforms of Facebook, Slack, and Reddit. These platforms were prioritized as they are different, popular media platforms and are commonly used by people from diverse backgrounds. Purposive sampling, as a non-random sampling technique is often used in targeted recruitment, because it is a feasible and efficient approach for pilot studies to ensure they recruit the subset of the population with the most relevant characteristics and experiences.

For recruitment, we first developed a list of keywords to identify COVID and/or long hauler groups (i.e., COVID-19, Coronavirus, long haulers, survivors, chronic, support, help, and recovery). Various combinations of these terms were utilized to identify potential social media groups from which participants could be recruited. The search was conducted, using the keywords, across a variety of platforms. In total, 16 Facebook groups, 1 Slack group, 1 Reddit subthread, and 2 organization websites were identified as targeting COVID-19 and/or COVID-19 long haulers. Six out of the 8 Facebook groups and the Slack group were private, meaning only those who were members of the group could see the post. The number of members in the Facebook groups ranged from 1,000 to 166,000 members.

Upon receiving approval from administrators of the groups and organizations, recruitment posts were shared to 8 Facebook groups, 1 Slack group, 1 Reddit subthread, and 1 organization’s website. The posts provided information on the aim of the work, brief introduction of the intervention, inclusion criteria, institutional affiliation, incentive for participation, and the contact information of the project coordinator, with an attached image of the study flyer. All participants completed an online informed consent form which explained the study purposes, voluntary nature, and confidentiality of the intervention. In the interest of privacy, participants were permitted to use separate Facebook accounts, which were independent of their personal accounts, if preferred. No personally identifiable information was collected throughout any research activities in the present study.

### Inclusion and exclusion criteria

**Fig 1:**
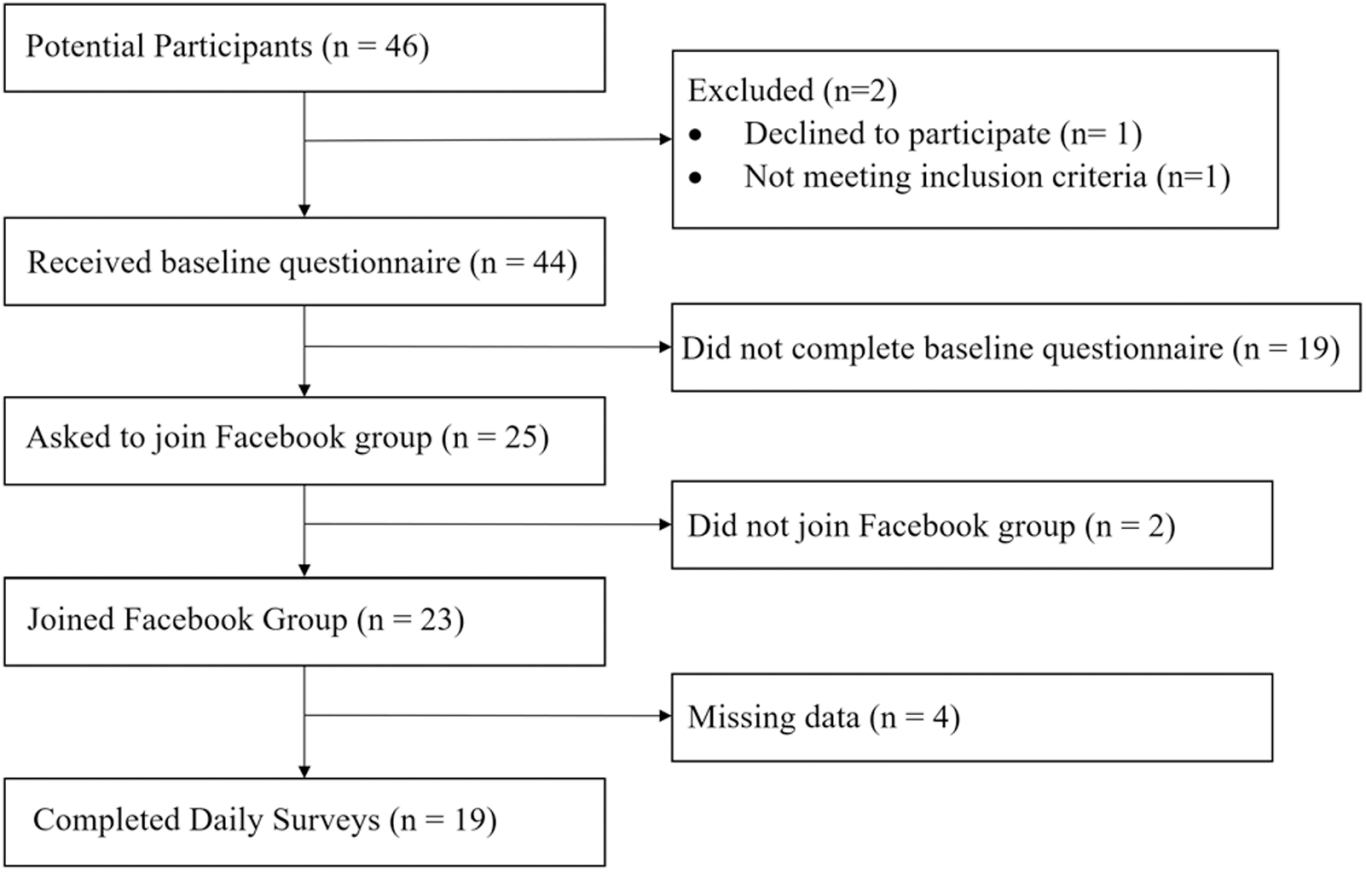
Participant screening.

In total, 46 individuals contacted the researcher, through email or Facebook Messenger, to participate in the study. They were eligible to participate in the study if they 1) were 18 years or older, 2) could speak and understand English, 3) had been infected with COVID-19, 4) had experienced at least one COVID-19 symptom for 4 weeks or longer after COVID-19 diagnosis, and 5) had no issues walking at a normal pace. Out of the 44 people who met the inclusion criteria, 23 people participated in the 4-week intervention (Fig 1). Those individuals who expressed interest but ultimately did not participate reported either a lack of interest or physical inability to participate.

### Mindfulness walking intervention

The MW sessions, adapted from Yang & Conroy, 2020 [18], were modified from conventional mindfulness meditation practices [34]. The sessions consisted of three primary practices: mindful breathing walking, step-focused walking, and full body scan walking. Mindful breathing walking focused on paying attention to the rhythm of the breath (i.e., each inhale and exhale). Step-focused walking focused on paying attention to the heel-to-toe rhythm that each foot follows as it contacts the ground. Full body scan walking focused on elevating attention to and awareness of each body part (e.g., top-down, bottom-up) to observe any emotional or physical sensations as they arise.

All MW sessions consisted of slow walking for 30 minutes, at a pace of approximately one step per second, at a convenient location in the participants’ neighborhood. Each successive week, the three mindfulness practices were progressively introduced and added to the slow walking session. The first session (week 1, session 1) did not include a mindfulness practice to allow the participants to practice slow walking for 30 minutes. The second session (week 1, session 2) included mindful breathing for only 5 minutes of the 30-minute slow walk. The third and fourth sessions (week 2) included mindful breathing walking for 10 minutes of the 30-minute slow walk. The fifth and sixth sessions (week 3) included 10 minutes of mindful breathing walking, followed by 10 minutes of step-focused walking during the 30 minutes slow walk. In addition to the mindfulness practices in the fifth and sixth sessions, the seventh and the eighth sessions (week 4) also included 10 minutes of full body scan walking during the last 10 minutes of the 30-minute slow walk.

### Implementation strategies and procedures

The intervention was implemented for two cohorts, wave 1 (n=12) and wave 2 (n=11). Wave 1 began in August 2021 and wave 2 began in November 2021. Participants were sent a baseline survey, via email, two weeks prior to the start of the intervention. Those who completed the survey were invited to join a private, intervention Facebook group. Using a private group protected participant privacy by limiting group membership to only the research team and participants. There were two separate Facebook groups for wave 1 and wave 2. Prior to the start of the MW sessions, participants were instructed to watch an orientation video, prerecorded by the research team, that briefly introduced the MW intervention, data collection, group rules, and provided contact information for technical support.

A prerecorded video that provided step-by-step instructions on that week’s MW session was uploaded to the intervention Facebook group every Monday. Participants could complete the week’s sessions at any time throughout the week, but a one-day gap was required between the two sessions, within a week. After each session, the intervention facilitator posted discussion prompts and questions to the group to promote MW adherence and to encourage the participants to share their experiences, questions, and feedback. The facilitators checked for and replied to the participants’ questions and comments each evening.

In addition to the MW sessions, participants were also asked to complete a daily diary survey, via RedCap, that was sent every evening of the 4-week intervention at 8 PM EST. To ensure response consistency, the survey was sent with instruction encouraging the participants to respond at the same time daily. The survey focused on participants’ emotional, mental, and physical wellbeing as well as their perceived mindfulness, in that moment. Questions about MW activities were also included in the daily survey to track MW session completion. In the one-month, post-intervention follow-up, the overall acceptability of the intervention was assessed using the Usefulness, Satisfaction, and Ease of Use Scale (USE), a valid and reliable instrument for subjective usability that can be applied to various scenarios of usability assessment because it is non-proprietary and technology-agnostic [35]. A raffle-based incentive was employed, where participants who completed the intervention had a chance to win one of six $30 USD e-gift cards.

### Ethics statement

The study protocol of recruitment and intervention was approved by the University of South Carolina Institutional Review Board (IRB # Pro00109358).

## Measures

### Baseline survey: Time-invariant covariates

#### Demographics.

The baseline survey included items on age, gender, race/ethnicity, education level, and state and county of residence.

#### COVID-19 related measures.

Key COVID-19 related metrics collected included COVID-19 diagnosis date, any hospitalization due to COVID-19, and pre-existing conditions before COVID-19 diagnosis. A variable for months since COVID-19 diagnosis was calculated by subtracting the diagnosis date from the demographics survey completion date.

### Daily diary survey: Time-varying covariates

#### Positive affect.

Positive affect was measured through three items: calmness (“How calm/relaxed are you today?”), energy (“How energetic/excited are you today?”), and happiness (“How happy/joyful are you today?”). The response options ranged from 1=not at all to 7=extremely. The sum score of these three items represented positive affect. These items were adapted from the “Positive Affect” measure in the Patient Reported Outcomes Measurement Information System (PROMIS) [36,37].

#### Negative affect.

Negative affect was measured through three items: stress (“How stressed are you today?”), loneliness (“How alone/lonely are you today?”), and fatigue (“How fatigued/tired are you today?”). The response options ranged from 1=not at all to 7=extremely. The sum score of these three items represented negative affect. These items were adapted from the “Negative Affect” measure in PROMIS [36,37].

#### Perceived cognition.

Perceived cognition was measured through two items: sharpness (“How sharp is your mind today?”), and concentration (“I can concentrate and keep track of things today.”). The response options ranged from 1=not at all to 7=extremely. The sum score of these two items represented perceived cognition. These two items were adapted from the PROMIS Cognitive Function - Abilities Subset (v2.0) to assess perceived cognition in real-time [38,39].

#### Mindfulness.

 Mindfulness was measured through two items from the State Mindfulness Scale (SMS) [40]. The SMS was originally developed to quantify situational mindfulness (i.e., mindfulness levels at a specific time and within a specific context). The overall SMS scale had sound internal consistency (α =.95), along with its two factors: Mindfulness of Body (α =.95) and Mindfulness of Mind (α =.90). Two items were selected to represent the two factors (i.e., Mindfulness of Body, Mindfulness of Mind). The first item belonged to the Mindfulness of Mind factor and measured emotional awareness (“I am aware of any thoughts or emotions in me today.”). The second item belonged to the Mindfulness of Body factor and measured physical awareness (“I am aware of any physical feelings or sensations from my body today.”). Both items had 7-point Likert scale response options ranging from 1=not at all to 7=extremely. The sum score of both items was used to represent mindfulness.

#### Physical activity minutes on any given day.

The physical activity levels of the participants, each day, were measured through a single item: “Approximately, how much time did you exercise today (not including the MW time)?”

#### MW on any given day.

The participants self-reported whether they practiced MW on a given day through a single item: “Did you practice mindful walking today?” The participants responded “Yes” or “No” to this item.

#### Days of practicing MW.

The total number of days a participant practiced MW was computed by adding up the “Yes” responses to “Did you practice MW today?”, for each participant separately.

#### Time-varying covariates at the day level.

Two time-varying covariates were assessed in the daily diary surveys, including if it was a typical day for the participant (“Do you feel sick or ill today?” (Yes/No), and their social engagement (“How many people did you talk to/interact with today?”). Also, “Time of finishing the survey,” (i.e., the time at which participants entered their daily survey responses) was automatically recorded by RedCap.

### One-month follow-up survey

#### USE scale.

The overall acceptability or the usefulness, satisfaction, and ease of use of the intervention was assessed using the USE scale at the one-month follow-up time point [35]. The scale consisted of 30-items with a 7-point Likert scale of response options that ranged from strongly disagree to strongly agree. The USE scale measured usefulness (8-items), ease of use (11-items), ease of learning (4-items), and satisfaction (7-items). An overall mean score of 4 or more was regarded as acceptable [35].

### Qualitative data collection

During the intervention implementation, qualitative data was extracted from the linguistic content posted by participants on the Facebook group page. Upon completion of the intervention, the research team member, who acted as the Facebook group administrator, checked and ensured that we collected all available linguistic data under each intervention session post. The linguistic data included comments, questions, reflections, and replies to the specific intervention materials (e.g., skill learning videos, reflection questions, and discussion posts). To protect the participants’ privacy, data was collected in a spreadsheet without any identifiable information (e.g., users’ names, sources of data). Two research team members synthesized the data using a pragmatic analytic approach to identify information associated with intervention feasibility (e.g., satisfaction) and efficacy evaluation (e.g., specific changes after practicing an intervention session). The themes identified by the two research members were compared and discussed during in-group meetings, to ensure reliability. Responses from the intervention facilitators (i.e., the research team) were excluded from the analyses.

## Data analysis

### Quantitative data analysis

Mean scores were calculated for the USE scale to evaluate the acceptability of the MW intervention. Multilevel models were used to analyze the data from the daily diary surveys, due to the nested structure of the data (i.e., days nested within participants). Data cleaning involved two important steps. First, if any participant answered a survey multiple times within a day, then only the last response by the participant was considered. Second, if any participant answered the daily prompt post-midnight, but before sleep time (i.e., any time before 3 AM), then that response was attributed to the previous day. Additionally, the distribution of each outcome variable, including positive affect, negative affect, perceived cognition, mindfulness, and physical activity, was examined. All variables except physical activity had a reasonable skewness and kurtosis between +0.5 and −0.5 [41]. Physical activity had a skewness and kurtosis of 2.61 and 10.53, respectively. As skewed data can lead to non-normal residuals, violating assumptions and reducing the validity of the statistical inferences, the Box-Cox transformation was applied to the outcome of physical activity minutes on any given day [42]. The Box-Cox transformation significantly reduced the skewness and kurtosis of the physical activity outcome to 0.11 and −1.83. The Box-Cox transformation therefore stabilized variance and brought the data closer to a normal distribution, improving model fit and interpretability. The transformed variable was used for analysis. Continuous covariates, including age and time of survey completion, were grand mean centered to avoid multi-collinearity issues and to improve interpretability [43].

Five multi-level models examined the association of the outcome variables on a given day (i.e., positive affect, negative affect, perceived cognition, mindfulness, physical activity), and their primary predictors (i.e., MW on a given day, total number of MW days across the study), after controlling for demographics and time-varying covariates. Each model included random intercept effects for a better model fit. The models were estimated using R 4.2.0 with the nlme package [44]. The final multilevel model was:

**Level-1** Positive Affect_ti_/Negative Affect_ti_/Perceived Cognition_ti_/Mindfulness_ti_/Physical activity_ti_ = β_0_ + β_1i_ (MW on a given day_ti_) + β_2i_ (Time of finishing the survey_ti_) + β_3i_ (Typical day_ti_) + β_4i_ (Social engagement_ti_) + e_ti_
**Level-2** ${\text{β}}_{0}=\gamma_{00}$ + $\gamma_{01}$ (Number of MW days_ti_) + $\gamma_{02}$ (Age) + $\gamma_{03}$ (Gender) + $\gamma_{04}$ (Race) + $\gamma_{05}$ (Education) + $\gamma_{06}$ (months since COVID-19 diagnosis) + $\gamma_{07}$ (history of COVID-19 hospitalization) + $\gamma_{08}$ (pre-existing medical conditions) + u_0i_

$$\beta_{\left(1-4\right)i}=\gamma_{\left(1-4\right)0}$$

where the level-1 equation constitutes time-varying covariates (i.e., variables that change daily), and the level-2 equation constitutes the time-invariant covariates (i.e., variables that do not change daily). Within the level-1 equation, β_0_ is the intercept, defined at level-2, β_(1–4)i_ are the estimated slope of the relationship between the outcomes and time-varying covariates, and e_ti_ is the momentary-level residual, capturing the unexplained variability for individual *i* at time *t*. Within the level-2 equation, the intercept of level-1 (β_0_) was defined as where: $\gamma_{00}$ is the overall mean, or the intercept; $\gamma_{01}\text{to }\gamma_{08}$ are the between-person associations between the outcomes and time-invariant covariates; and u_0i_ is the person-specific residual deviations, or random intercepts for the four outcomes, in the respective models. The equation, β_(1–4)i_ = $\gamma_{\left(1-4\right)0}$, indicates that no random slope effect was added for β_(1–4)i_, based on the assumption that the relationships between the time-varying predictors and the outcomes were consistent across individuals, primarily due to the limited sample size.

### Qualitative data analysis

Paradata was analyzed using a pragmatic approach, which has been widely used in mixed methods studies [45]. This was performed by: (1) extracting data from the Facebook group page and familiarizing ourselves with the data; (2) generating a coding scheme; (3) structuring the data and identifying emergent themes; (4) reviewing the coded data and comparing patterns; and (5) defining themes and their interpretations [45]. Descriptive statistics, such as the frequencies and percentages of posts that corresponded to a theme, were also calculated. The structure of the themes and dimensions are presented in the S1 Appendix.

## Results

### Participant demographics

Out of the 23 participants who engaged in the study activities, 19 participants were included in the analysis due to missingness from the remaining 4 participants. The demographic information of the included (n=19) and excluded (n=4) participants is reported in Table 1. The mean age of the participants was 47.37 years (SD=11.58). A majority of participants were Caucasian (*n*=16, 84%), women (*n*=17, 89%), and had completed a bachelor’s degree (e.g., BA, BS) (*n*=9, 47%). Most participants reported pre-existing conditions before the COVID-19 diagnosis and only 3 (16%) had been hospitalized due to COVID-19.

**Table 1:** Participant characteristics (*n* = 23).

	Participants included in the analysis (n=19)		Participants excluded due to missingness (n=4)	
	*n* or *Mean*	*%* or *SD*	*n or Mean*	*% or SD*
**Age** (*Mean*, *SD*)	47.37	11.58	44.5	16.94
**Gender** (*n, %)*				
Women	17	89.47%	4	100%
Men	2	10.53%	–	–
**Race/ethnicity** (*n, %)*				
Caucasian	16	84.21%	3	75%
Black/African American	2	10.53%	1	25%
Other	1	5.26%	–	–
**Education level** (*n, %)*				
High school degree or equivalent	1	5.26%	–	–
Some college, but no degree	3	15.79%	1	25%
Associates degree (e.g., AA, AS)	1	5.26%	1	25%
Bachelor’s degree (e.g., BA, BS)	9	47.37%	1	25%
Post-graduate degree or above (e.g., Master’s, Ph.D., MD, JD)	5	26.32%	1	25%
**Current residence** (*n, %)*				
Florida	–	–	1	25%
Missouri	–	–	1	25%
Utah	–	–	1	25%
California	1	5.26%	–	–
Georgia	1	5.26%	–	–
Illinois	1	5.26%	–	–
Iowa	1	5.26%	–	–
Kentucky	1	5.26%	1	25%
Louisiana	1	5.26%	–	–
Maryland	1	5.26%	–	–
Minnesota	1	5.26%	–	–
New Jersey	1	5.26%	–	–
New York	1	5.26%	–	–
North Carolina	2	10.53%	–	–
Ohio	1	5.26%	–	–
South Carolina	3	15.79%	–	–
Texas	1	5.26%	–	–
Washington	2	10.53%	–	–
**Ever been hospitalized due to COVID-19** (*n, %)*				
No	16	84.21%	3	75%
Yes	3	15.79%	1	25%
**Pre-existing conditions before COVID-19 diagnosis** (*n, %)*				
No	7	36.84%	2	50%
Yes	12	63.16%	2	50%
**Months since COVID-19 diagnosis** (*Mean*, *SD*)	10.43	4.20	11.52	2.43

*SD* = *Standard Deviation.*

### Feasibility and acceptability

The feasibility of the MW intervention was assessed through survey responses and the number of participants practicing MW. A high response rate to the daily diary surveys indicated good adherence to the study activities. A high number of days practicing MW reflected the ability and actions of participants implementing the intervention activities. Of the 28 total daily diary surveys, participants responded to nearly 16 surveys, on average (mean=15.9, SD=9.2). The response rate to the daily diary surveys, for each participant, ranged from 7% to 96%, with a mean of 57% (SD=33%). Participants with a low response rate were not excluded from the analysis because even data from a single day could provide crucial information to assess the day-to-day impact of MW on mental health outcomes. The total number of days participants practiced MW ranged from 0 to 24, with a mean of 7.29 days (SD=5.25). On average, participants practiced MW on 1.72 days (SD=1.38) of a given week.

Both qualitative and quantitative data were used to assess acceptability. The Paradata (i.e., participants’ posts) showed that participants were satisfied with the intervention in terms of mindfulness skill enhancement (n=11; i.e., attention, mindful breathing, slow walking), pain reduction (n=1), psychological wellbeing promotion (n=2; i.e., emotion regulation and stress relief), and willingness to participate in similar interventions (n=2) (S1 Appendix). In the one-month follow-up survey, the USE scale was used to measure acceptability. Within a range of 1–7, where higher scores indicate a higher acceptability in that aspect, the mean scores of the key USE subscales were: usefulness=4.57 (SD=2.02), ease of use=5.04 (SD=1.57), ease of learning=5.44 (SD=1.87), and satisfaction=4.69 (SD=1.43). The overall acceptability (i.e., the overall mean score) was 4.93 (SD=1.88). An overall mean score of 4 or more was regarded as acceptable.

### Preliminary efficacy

Table 2 shows the descriptive statistics of the primary variables and time-varying covariates. A total of 303 responses were collected from 19 participants over the course of the intervention. Participants practiced mindful walking for an average of 7.29 days (SD=5.25) out of 24 days. Beyond time spent completing the MW sessions, participants engaged in an average of 19.86 minutes (SD=27.56) of additional physical activity on any given day. After practicing MW, participants reported moderate levels of positive affect (M=3.95, SD=1.14), lower levels of negative affect (M=3.33, SD=1.26), and higher levels of perceived cognition (M=4.38, SD=1.30) and mindfulness (M=5.29, SD=1.17).

**Table 2:** Descriptive statistics for primary variables and time-varying covariates (n=19, 303 rows).

	N or Mean	% or SD
Main variables (*Mean*, *SD*)		
Positive affect (3 items, scale 1−7)	3.95	1.14
Negative affect (3 items, scale 1–7)	3.33	1.26
Perceived cognition (2 items, scale 1–7)	4.38	1.3
Mindfulness (2 items, scale 1–7)	5.29	1.17
Physical activity (mins on a given day)	19.86	27.56
Number of days engaged in MW (*Mean*, *SD*)	7.29	5.25
MW on any given day (*n*, *%*)		
No	198	65.56%
Yes	104	34.44%
Number of MW sessions practiced (*n*, *%*)		
Session 1 (week 1)	18	17.14%
Session 2 (week 1)	17	16.19%
Session 3 and 4 (week 2)	27	25.71%
Session 5 and 6 (week 3)	27	25.71%
Session 7 and 8 (week 4)	16	15.24%
Time of daily diary survey completion (*n*, *%*)		
Before 8 AM	9	2.97%
08–10 AM	8	2.64%
10–12 PM	8	2.64%
12–02 PM	10	3.3%
02–04 PM	5	1.65%
04–06 PM	2	0.66%
06–08 PM	–	–
08–10 PM	261	86.14%
10–12 Midnight	9	2.97%
Typical day (*n*, *%*)		
No	186	61.39%
Yes	117	38.61%
Social engagement (Number of people) (*Mean*, *SD*)	10.15	12.59

Table 3 presents the results of the multilevel model analysis regarding the preliminary efficacy of the MW intervention, in terms of mindfulness, positive affect, negative affect, perceived cognition, and physical activity, when controlling for day-level and demographic covariates (i.e., age, gender, race/ethnicity, education, months since COVID-19 diagnosis, hospitalization due to COVID-19, pre-existing medical conditions, time of survey completion, typical day, and social engagement levels on that day). The independent variables included whether the participant practiced MW on any given day and the total number of days they engaged in MW. The findings on the preliminary efficacy of the intervention were promising. Specifically, MW uptake on any given day was significantly associated with higher positive affect (*β*=0.89, *p*<0.01), lower negative affect (*β*=−0.83, *p*<0.01), higher perceived cognition (*β*=0.52, *p*<0.05), and higher physical activity levels (*β*=0.41, *p*<0.05). The total number of MW days, across the study period, were significantly and positively associated with higher mindfulness levels (*β*=0.3 *p*<0.01).

**Table 3:** Multilevel model results (n=19, 303 rows).

	Positive Affect		Negative Affect		Perceived Cognition		Mindfulness		Physical Activity	
	Value	SE	Value	SE	Value	SE	Value	SE	Value	SE
***Fixed Effects***										
(Intercept)	9.3***	2.02	10.1***	2.52	5.79**	1.78	3.63*	1.55	0.53	1.03
**MW on any given day** (No=0, Yes=1)	0.89**	0.32	−0.83**	0.31	0.52*	0.25	0.03	0.19	0.41*	0.16
**Total MW days**	0.13	0.12	−0.08	0.15	0.04	0.1	0.3**	0.09	0.06	0.06
**Age**	0.03	0.04	−0.14*	0.05	0.02	0.04	−0.04	0.03	−0.02	0.02
**Gender** (Men) Reference: Women	−4.79*	1.8	1.34	2.32	−1.79	1.61	−2.51	1.42	−0.34	0.92
**Race** (Caucasian) Reference: Other	1.29	1.31	0.77	1.67	0.94	1.17	2.71*	1.02	−0.72	0.67
Education	0.38	0.49	−1.2	0.62	0.52	0.44	1.11*	0.38	0.35	0.25
**Months since COVID-19 diagnosis**	−0.23	0.16	0.42	0.2	−0.07	0.14	−0.23	0.13	−0.09	0.08
**COVID-19 hospitalization** (No=0, Yes=1)	1.08	1.56	0.1	2.02	1.06	1.4	−0.08	1.23	1.43	0.8
**Pre-existing medical conditions** (No=0, Yes=1)	4.32**	1.29	−3.51	1.68	2.09	1.16	3.3**	1.03	1.42	0.65
**Time of survey completion**	−0.08*	0.03	0.06	0.03	−0.02	0.03	0.01	0.02	0	0.02
**Typical day** (No=1, Yes=0)	−2.61***	0.37	2.57***	0.36	−1.73***	0.29	−0.24	0.23	−0.22	0.19
**Social engagement** (No=0, Yes=1)	0.02	0.01	0.01	0.01	0.02*	0.01	0.01	0.01	0.02**	0.01
***Random Effects***										
Intercept variance	2.76		5.06		2.31		1.88		0.72	
Residual variance	5.48		5.11		3.46		2.04		1.39	

SE= Standard Error

***p <.001;

**p <.01;

*p <.05.

Of the posts made by participants, most included messages or dialogue about the benefits of participating in the intervention program. The benefits included “awareness/mindful enhancement”, “emotional change”, and physical wellbeing improvements. In terms of “awareness/mindful enhancement”, participants noted that the MW sessions promoted their ability to be present in the moment and to be aware of their bodily changes, in several aspects. The changes included “physiological awareness” (e.g., “*I am finding it easier to mindfully walk while paying attention to my breath*”), “behavioral awareness” (e.g., “*I realized walking one step per second was actually difficult*”), and “self-consciousness/focus on the self” (e.g., “*Now I notice that when people walk by me, I’m still focusing on my steps*”). One participant mentioned an emotional change, noting that they felt pleasant when practicing MW, when they said: “*While walking this evening, I notice[d] the smell of fresh cut grass. Haven’t been something that I’ve really paid much attention to. It was refreshing*”. Participants noted that they experienced physical enhancement during the intervention. Physical enhancement involved “COVID-19 symptom relief” (e.g.,“*I was unaware that I was walking one step per second with my post COVID symptoms…During the session, I wasn’t concentrating on it—I suppose I knew it [COVID symptoms] was there, but the session distracted me from it”)* and “physical comfort” (e.g., “*Now I feel more comfortable*”).

### Challenges and limitations

Participants mentioned several challenges that arose throughout the intervention program. Some challenges were related to environmental factors, such as “bad weather” (e.g., “*It was bad weather that day…I got overwhelmed by paying attention to my breathing*”) and “natural disaster” (e.g., “*Unfortunately, my neighborhood is flooded, and I will be walking here this week”*). Participants also noted challenges to their engagement, based on aspects of the intervention program, including “difficulty learning MW skills” (e.g., “*I thought this last exercise was more difficult than the others. I felt like I was more focused on getting the skills correct than just being present and in the moment*”) and “negative emotions arising from MW practice” (e.g., “*Truthfully, I got overwhelmed by paying attention to my breathing. It felt like I had covid again*”).

## Discussion

### Principal findings

The current study tested the feasibility, acceptability, and preliminary efficacy of digitally delivering a MW intervention to long haulers via the social media platform Facebook, by using a mixed methods design that included daily diary surveys. Our findings demonstrate strong feasibility in terms of *adherence*, based on our low dropout rates, reasonable response rate to the daily diary surveys, and high engagement with the weekly MW sessions throughout the study period; and *acceptability*, based on our higher-than-average score on the USE scale (4.9/7), which demonstrates the participants’ perception of the intervention’s strong usefulness, ease of use, ease of learning, and satisfaction. In terms of preliminary efficacy, the multilevel analysis, using the daily diary surveys, indicated that on days participants engaged in MW, they reported increased positive affect, perceived cognition, and physical activity levels, and reduced negative affect. Additionally, the total number of days the participants engaged in a MW practice, across the study period, was linked to enhanced mindfulness on any given day.

The quantitative findings on the preliminary efficacy of the intervention were further supported by the qualitative findings as participants reported improved physical comfort through symptom relief, feeling pleasant when practicing MW, as well as physiological-, behavioral-, and overall self-awareness. Findings related to the intervention’s feasibility were enriched through understanding two factors influencing participation in MW sessions, which included external environmental factors (e.g., bad weather, flooding) and difficulties with learning the required MW skills. Overall, participants reported not only improved mental wellbeing but also experienced an increase in their physical comfort and physical activity levels on any given day.

### High feasibility and acceptability

The high feasibility of our MW program was indicated by low dropout rates, reasonable responsiveness to the daily diary surveys, and high engagement with the MW sessions. All 23 participants who joined the Facebook group committed to the 4-week MW program, with only 4 showing missingness in the daily diary surveys. The sustained participation of most participants in the MW program was evidenced by their ongoing involvement, indicating a strong interest in such interventions among long haulers. Participants responded to daily diary surveys about 60% of the time (SD=33), which aligns with previous EMA research among long haulers [46,47] and demonstrates a substantial commitment to the intervention’s daily self-monitoring.

Across the MW sessions, participants showed the highest engagement in week 2 (i.e., sessions 3 and 4; n=27, 25.71%) and week 3 (i.e., sessions 5 and 6; n=27, 25.71%). Each of these highly engaged sessions included 10 minutes of mindful breathing walking, with sessions 5 and 6 also including an additional 10 minutes of step focused MW. The lowest engagement occurred in week 4 (i.e., sessions 7 and 8; n=16, 15.24%), which included an additional 10-minute full body scan walking. It is notable that participants reported difficulty in learning the “last exercise” (i.e., a full body scan walking). Alternatively, within week 1, session 1 (n=18, 17.14%) and session 2 (n=16, 16.19%), which either included no mindful breathing or only 5 minutes of mindful breathing, respectively, had lower engagement. These findings suggest a preference for a balanced approach of mindful breathing and step-focused walking during the 30-minute slow MW, among long haulers.

The MW session instructions were released to the Facebook group at the start of each week and encouraged participants to practice each session at least once, aiming at two sessions per week. For instance, in week 1 participants were expected to practice sessions 1 and 2. Given the participation expectations, participants engaged in MW for an average of 1.72 days per week (SD=1.38), which roughly aligns with the expectation of two sessions weekly. Over the study period, participants practiced MW for an average of 7.29 days (SD=5.25), which is close to the anticipated participation in 8 sessions.

The average level of acceptability, measured using the USE scale (4.9/7), demonstrated participants’ perceptions of the intervention’s strong usefulness, ease of use, ease of learning, and satisfaction. Participants felt the MW was helpful overall. For instance, one participant said: “I’ve found this has helped me so much and been life changing because I had been on the go this last decade it seems like and this has brought me right back to where I need to be.” Some participants, however, reported difficulties in learning the mindfulness exercises, especially the walking full body scan, the last exercise. As one participant reported, “Of the three parts, I am still struggling with the body scans.” Overall, our results highly complement previous MW intervention studies where older adults [33], breast cancer patients [48], and community residents [49] have also reported high acceptability of MW interventions. Despite these promising results, the fluctuating engagement across sessions suggests a need for personalized motivational strategies and consideration of potential physical limitations, to further enhance future program engagement [50].

### Improved mental wellbeing and physical activity levels

The MW intervention demonstrated an improvement in participants’ mental wellbeing including heightened positive affect, perceived cognition, mindfulness, and reduced negative affect. The intervention also resulted in an increase in participants’ daily physical activity levels beyond the duration of the MW sessions. Participants reported, “It was a huge relief to get to my head so I could focus on clearing all thoughts of pain. Between enjoying being outside and clearing my thoughts, it did take my mind off of the pain temporarily. I am definitely going to try this again tomorrow.” Further, a participant said: “By the end of week 2, I was hooked. My perspective of walking was changing and no matter how bad I felt that day, I looked forward to getting off of work and seeing what new experience I would discover while walking down the same path and road I have walked for 30 years. While I do still struggle with mind focus on the body while walking, hopefully one day, the pain will ease and I will be able to gain a different experience from this as well.”

The findings of the present study are aligned with previous MW intervention research that has demonstrated similar findings among older adults [33,39], breast cancer survivors [48], college students [51], psychologically distressed individuals [23], general adults [52], young adults [53], and among adults with inadequate physical activity levels [54]. The Psychoneuroimmunological Framework offers insights into the observed outcomes. In alignment with the framework, MW induces a relaxation response, reduces chronic inflammation in long haulers [55], promotes attention regulation [56], counters cognitive challenges like ‘brain fog’ [56], fosters bodily awareness and interoception, aids in symptom management [57], releases endorphins that aid in pain relief and mood elevation [58], and promotes emotion regulation and neuroplasticity, which leads to improved emotional resilience [59].

Similarly, the present findings on the effects of MW on physical activity levels are also supported by previous MW intervention research. For example, breast cancer patients increased physical exercise due to MW [48]. Adults with inadequate levels of physical activity have exhibited significant improvements in physical activity after engaging with MW [54]. Further, patients with chronic obstructive pulmonary disease also reported improvements in their exercise capacity because of engaging in MW [21]. The present work offers preliminary findings that suggest that MW could be a viable approach for improving physical activity among long haulers. The low-intensity nature of MW makes it an accessible exercise with the ability to aid in rebuilding physical strength and endurance without excessive exertion [21–23]. A unique contribution of the present work to existing literature is the exploration of the mental and physical health benefits of a MW intervention digitally delivered to long haulers. Therefore, based on these encouraging results, this work strongly advocates for the adoption of MW interventions in chronic condition management, such as long COVID.

### The need for online interventions

Our intervention uniquely harnessed the power of social media, as it hosted each phase of the study from recruitment to evaluation. With the increasing penetration of smartphones and internet access across the globe, online interventions hold promise for wide-reaching impact, making quality care accessible to broader populations [60]. Given these preliminary findings on the feasibility of delivering a MW intervention, via social media, future randomized controlled trials (RCTs) should be conducted to evaluate the scalability and affordability of such online interventions, especially in low-income settings. For instance, a tailored study could be designed to target long haulers in the rural communities of sub-Saharan Africa, where, despite infrastructure challenges, mobile connectivity has surged significantly [61,62]. Future RCTs and tailored studies could offer insights into potential intervention barriers and facilitators, specific to resource-limited settings, such as local digital literacy levels [63] or cultural perceptions of online health interventions [64]. Moreover, studies should also consider integrating vernacular language and culturally relevant content to enhance engagement and relatability [64].

The time and context in which an MW intervention is implemented is essential to consider. For example, cultural perceptions of social media and mindfulness may influence the acceptance of digital interventions. Additionally, a lack of accessible infrastructure (e.g., parks, public green lands) for physical activity may impede conducting and engaging in MW interventions. Ultimately, assessing the cost-effectiveness and applicability of digitally delivered interventions in resource-limited settings, where healthcare resources are often limited, is vital [50,65].

### Social engagement as a catalyst

While MW is typically an individual activity, delivering our MW intervention through a Facebook group leveraged social engagement. The social nature of a Facebook group created a sense of community, where participants could actively engage with one another and the research team through comments and posts. Within the group, participants could post photos and videos of their walking, share their experiences participating in the MW intervention, and ask questions about MW skills and intervention logistics. Exemplifying the therapeutic potential of online social groups for mutual support in pain and recovery [66], participants shared their experiences and motivated each other through the group. The group played an essential role in maintaining motivation and commitment to the intervention by enabling participants to watch their peers’ progress. Among long haulers who often feel isolated, the group also had the potential to enhance participants a sense of belonging, which is crucial for emotional wellbeing [67,68]. For instance, one participant left comments like, “This is now one of my favorite groups to be a part of. Once again, thanks for allowing me to join and participate. Let me know if you come up with another study, I would love to help out.” Thus, the impact of the intervention extended beyond solely physical and mental benefits, as it also included socio-emotional benefits, through shared experiences.

Future research should further explore the impact of online social groups, particularly in collectivist societies where community and social connections ties are vital [69]. Online interventions that emphasize collective healing, shared experiences, and mutual support can be particularly impactful. Future studies should investigate how tailored interventions, within varying contexts, could leverage cultural values and social structures to enhance the therapeutic potential of online groups. Understanding virtual community dynamics within specific societal contexts could facilitate the creation of effective and culturally relevant interventions that are tailored to address the unique needs of individuals, within a variety of cultures [70].

### Strengths and limitations

A key strength of our study was the use of social media in all study activities, from recruitment to evaluation. The online intervention was especially effective during the COVID-19 pandemic as it permitted safe, remote participation. Secondly, as related to the quantitative assessments, our study used daily diary surveys, which offered dynamic intervention monitoring and nuanced insights into its day-to-day impact, providing a more granular understanding that is often missed in retrospective assessments [71]. Moreover, the extraction of Paradata from the Facebook group added a rich layer to our findings. Paradata, in this context, captured the lived experiences of participants, their spontaneous reactions, and organic discussions, thereby providing an unfiltered perspective of the intervention’s effects and potential areas of improvement [35].

The study, while promising, also presents several limitations. First, the sampling methods used (i.e., purposive yielded a sample of participants that were primarily Caucasian women with high education attainment, limiting the generalizability of our findings because this subgroup might not represent individuals with other demographic and socioeconomic backgrounds. Relatedly, the recruitment of participants exclusively from social media might introduce selection bias, representing a subset of long haulers who are more digitally active, who have a higher digital literacy, or who are more so inclined to join such groups. Therefore, future studies that include the experiences and perspectives of diverse racial and ethnic groups or with different pre-existing chronic conditions are necessitated. Further, the collection of only daily diary survey data restricts our understanding of the intervention’s prolonged effects and informs our call for future studies that apply a longitudinal approach, including extended follow-ups. Additionally, the absence of a control group in the study design limits our ability to definitively attribute observed outcomes to the intervention. RCTs are needed to assess the efficacy of MW intervention through a rigorous study design. Finally, while Paradata from the Facebook group offers valuable insights, it might not capture the full range of participants’ experiences. Future studies should conduct in-depth interviews to gather more reliable information.

## Conclusions

Overall, the present study demonstrated the feasibility, acceptability, and preliminary efficacy of a digitally delivered (i.e., Facebook-based) MW intervention for long haulers, through a mixed methodology approach. Positive findings related to intervention adherence and acceptability reflect the suitability of digitally delivering MW interventions. The benefits of the intervention are demonstrated as participants reported enhanced positive affect, perceived cognition, and physical activity, along with reduced negative affect on the days they engaged in MW. Additionally, the total number days participants engaged in MW was linked to mindfulness on any given day. These encouraging outcomes strongly advocate for the adoption of MW interventions for the management of chronic conditions such as long COVID. Given the accessibility and low resource demands of online delivery, this intervention has the potential to be particularly beneficial in contexts where there are barriers to traditional healthcare delivery (e.g., rural communities, communities experiencing health disparities). Digitally delivered MW interventions offer a cost-effective strategy to cope with the burden of long COVID and to improve both the physical and psychosocial wellbeing of long haulers.

## Supporting information

S1 Appendix Results of qualitative analysis of Paradata from Facebook posts (*n* = 28). (DOCX)

S1 Data Quantitative data of the study. (SAV)
