# Supplementary material for: Feasibility and preliminary efficacy of an online mindful walking intervention among COVID-19 long haulers: A mixed methods study including daily diary surveys
Source: PLOS Digit Health. 2025 Apr 8;4(4):e0000794. doi: 10.1371/journal.pdig.0000794 (PMC11978064; doi:10.1371/journal.pdig.0000794)
Supplement: S1 Appendix — (DOCX) [file pdig.0000794.s001.docx]

S1 Appendix. Results of qualitative analysis of paradata from Facebook posts (*n* = 28)

| **Themes** | **Example quotations** | **N of posts** | **%** |
| --- | --- | --- | --- |
| **Positive influences of the intervention program** | | | |
| ***Awareness/mindfulness enhancement*** | |  |  |
| Physiological awareness | “I am finding it easier to mindfully walk while paying attention to my breath. I seem to find a rhythm whereas just walking I would pick up the pace every so often. Changed my scenery a bit today as I am located elsewhere. That helped a lot too” | 7 | 25% |
| Behavioral awareness | “I am finding it easier to mindfully walk while paying attention to my breath. I seem to find a rhythm whereas just walking I would pick up the pace every so often. Changed my scenery a bit today as I am located elsewhere. That helped a lot too” | 9 | 32% |
| Self-consciousness | “Yes! The more I do it, the more I feel like I am paying attention to my steps. In the beginning, I would get self-conscious if someone walked by me. I would stop my meditation and walk normally until I was out of their sight. Now I have noticed that when people walk by me, I'm still focusing on my steps.” | 2 | 7% |
| ***Physical enhancement*** | |  |  |
| COVID-19 symptom relief | “I was unaware that I was walking one step per second with my post covid symptoms. These symptoms are nerve pain in my feet, achy knees, and a cough from doing any type of physical activity. During the session, I wasn't concentrating on it-I suppose I knew it was there, but the session distracted me from it. In a way, I almost felt like I was doing an activity pre-covid. I did feel those symptoms afterward though.” | 1 | 4% |
| Physical enhancement | “Now I feel more comfortable.” | 1 | 4% |
| ***Emotional change*** | |  |  |
| Feeling pleasant | “While walking this evening, I noticed the smell of fresh cut grass. Hasn’t been something that I’ve really paid much attention to. It was refreshing.” | 1 | 4% |
| Challenges when implementing the intervention program | | | |
| ***Environmental*** | | | |
| Bad weather | “I found it really hard to pay attention to my breathing. I had to stop in the middle of it because I got light headed and had shortness of breath. It was bad weather that day, so I'm sure that didn't help it. Truthfully, I got overwhelmed by paying attention to my breathing. It felt like I had covid again.” | 3 | 10% |
| Natural disaster | “Unfortunately, my neighborhood is flooded and I will be waking here this week. I will walk early morning or later in the evening. Louisiana summers are brutal.” | 1 | 4% |
| ***Aspects of the intervention*** | | |  |
| The difficulty of learning mindful walking skills | “I thought this last exercise was more difficult than the others. I felt like I was more focused on getting the skills correct than just being present and in the moment.” | 1 | 4% |
| Negative emotion arising from mindful walking practice | “Truthfully, I got overwhelmed by paying attention to my breathing. It felt like I had covid again.” | 1 | 4% |
|  |  |  |  |
